# Social media use and the not-so-imaginary audience: Behavioral and neural mechanisms underlying the influence on self-concept

**DOI:** 10.1016/j.dcn.2021.100921

**Published:** 2021-01-26

**Authors:** S. Peters, R. Van der Cruijsen, L.P.E. van der Aar, J.P. Spaans, A.I. Becht, E.A. Crone

**Affiliations:** a Department of Developmental Psychology, Leiden University, Wassenaarseweg 52, 2333AK, the Netherlands; b Leiden Institute for Brain and Cognition, The Netherlands, Albinusdreef 2, 2333 ZA, Leiden, the Netherlands; c Erasmus School of Social and Behavioural Sciences, Erasmus University Rotterdam. Burgemeester Oudlaan 50, 3062 PA, Rotterdam, the Netherlands; d Research Center Adolescent Development, Utrecht University. Heidelberglaan 8, 3584 CS Utrecht, the Netherlands

**Keywords:** Social media, Self-concept, Medial prefrontal cortex, Adolescence, Self-esteem, Development

## Abstract

We investigated behavioral and neural mechanisms in the relation between social media use (SMU) and self-concept, as well as longitudinal developmental outcomes. Adolescents and young adults (*N* = 150, 11–21 years old at T1) rated themselves on 60 traits in the academic, physical and prosocial domain, and also indicated how they thought peers would judge them (reflected-peer-judgements). Longitudinal questionnaires (1- and 2-year follow-up) were collected to assess positive (prosocial behavior, self-concept clarity) and negative (clinical symptoms) long-term outcomes.

Results indicated that heavier self-reported SMU was linked with lower difference scores between self-judgements and reflected-peer-judgements. Lower SMU was related to more positive ratings from self-judgements vs. reflected-peer-judgements. SMU was also associated with less positive self-concept, particularly in the academic domain (boys and girls) and physical domain (girls). Neurally, increased SMU was linked to heightened mPFC-activity during self-judgements compared to reflected-peer-judgements, and increased activity during physical compared to academic and prosocial self-judgements. Longitudinal analyses indicated no evidence for long-term effects of social media use, self/reflected-peer-difference scores and mPFC-activity on clinical symptoms, prosocial behavior or self-concept clarity. This study highlights the complex relationship between social media use and wellbeing and future research is needed to confirm the lack of long-term effects.

## Highlights

• Our findings reveal that adolescents with increased social media use (SMU) showed:
• Lower difference scores for self-ratings vs. how they expected peers would rate them.
• Higher mPFC-activity for self-ratings vs. reflected-peer-ratings.
• Higher mPFC-activity for physical self-ratings vs. academic/prosocial self-ratings.
• Less positive self-concept, but no convincing link with future clinical symptoms.

## 1 Introduction

Social media use (SMU) is rapidly becoming omnipresent, especially amongst adolescents (Anderson and Jiang, 2018). Compared to offline communication, social media create an environment with more prominent opportunities for self-presentation, peer-feedback on the self and social comparison (Valkenburg, 2017). Adolescents are highly sensitive to social evaluation (Somerville, 2013), which affects their self-concept development (Sebastian et al., 2008) in a time during which self-concept is still undergoing major changes (Harter, 2015). Therefore, it is important to consider whether and how SMU affects self-concept in adolescence.

Prior work on whether SMU affects self-concept (the estimated qualities and traits of the self) or the related construct of self-esteem (a feeling of self-worth) are contradictory. Several studies reported negative effects, i.e. an association between increased SMU and lower self-esteem (Woods and Scott, 2016) and a connection between emotional investment in social network sites and reduced self-esteem (Blomfield Neira and Barber, 2014). Other studies point towards positive effects, such as a link between socializing on social media and higher self-esteem (Apaolaza et al., 2013), and higher self-esteem after viewing one’s own Facebook profile (Gonzales and Hancock, 2011).

Prior work has established that self-concept can be described not only globally, but also in specific domains, with the most important distinctions being the physical, academic and social domain (Harter, 1988). Several studies suggested that especially physical self-concept might be negatively influenced by SMU, as personal photos and physical appearance play a large role on social network sites, and people usually present idealized images of themselves online (de Vaate et al., 2018). For the social domain, SMU appears mostly associated with positive effects on social self-esteem (Blomfield Neira and Barber, 2014; Valkenburg et al., 2017). Insights into the behavioral and neural mechanisms of not only whether, but also why SMU relates to overall and domain-specific self-concept might contribute to unraveling the current contradictory evidence.

One pathway through which social media may be related to self-concept is by influencing how adolescents reflect upon their own traits. Potentially, being active on social media may enhance the feeling of constantly being judged by peers that adolescents typically experience – a phenomenon known as the ‘imaginary audience’ (Elkind, 1967). Heavier SMU might lead adolescents to more frequently view themselves with the judgements of peers in mind. We therefore examined whether SMU is associated with self-judgements, and how they think same-aged peers will judge them (‘reflected-peer judgements’). Specifically, we calculated how much more positive the adolescent's self-judgements were compared to these reflected-peer-judgements. We expected that this difference would be smaller with heavier social media use. Additionally, we analyzed whether sex played a moderating role in the relationship between social media use and self-concept. Previous work on the relation between SMU and wellbeing in general indicated that, although effects of social media on wellbeing were very small, they might be slightly stronger in female participants (Orben et al., 2019).

Another pathway through which social media might be related to self-concept is through the neural processing of self- and reflected-peer-judgements. The brain regions underlying self-processing have been extensively studied. The core region in this network is the medial prefrontal cortex (mPFC) (Denny et al., 2012). Importantly, mPFC is still undergoing structural and functional development during adolescence (Burnett et al., 2011; Mills et al., 2012). Prior studies revealed that mPFC-activity peaks in adolescence relative to childhood and adulthood while being observed by others (Somerville et al., 2013; Van Hoorn et al., 2016). Tentatively, this may make adolescents especially sensitive to the frequent peer-judgments through social media. Intriguingly, prior research demonstrated a strong overlap in mPFC for self-judgements and reflected-peer judgements (van der Cruijsen et al., 2019). This demonstrates that mPFC is also implicated during reflected judgements, i.e. rating yourself from the perspective of others (Pfeifer et al., 2009). We therefore employed fMRI-measures to appraise whether heavier SMU was associated with altered mPFC-activity during self-judgements and reflected-peer-judgements.

Finally, it is a highly debated question whether SMU influences developmental outcomes, and if so, whether the influence is positive or negative. We therefore also tested for longitudinal relations between SMU, mPFC-activity and self/reflected-peer-difference with future positive and negative outcomes. We investigated internalizing and externalizing clinical symptoms, which have already been linked to SMU in prior research (Booker et al., 2018; McCrae et al., 2017). For positive effects, we assessed prosocial behavior and self-concept clarity. Self-concept clarity refers to the stability rather than the content of the self-concept (e.g. “I have a clear idea of who I am”). We hypothesized that SMU might encourage acting prosocially (Boulianne et al., 2018) and might lead to greater self-concept clarity through e.g. frequent social feedback and expanded opportunities to experiment with different versions of yourself (Valkenburg, 2017). To examine the longitudinal associations between SMU, prosocial behavior and self-concept clarity we used questionnaire follow-up data from the second (T2) and third timepoint (T3) approximately 1 and 2 years later.

Taken together, we studied adolescent’s behavioral responses and mPFC-activity during self-judgements and reflected-peer-judgements in the physical, academic and prosocial domain (van der Cruijsen et al., 2017). We hypothesized that SMU would be associated with 1) less positive self-concept, 2) less difference between self-judgements and reflected-peer-judgements, and 3) that this would be mirrored in less difference in mPFC-activity during self-judgements and reflected-peer-judgements. Finally, we tested for longitudinal relations between SMU and self/reflected-peer-difference and future clinical symptoms, prosocial behavior and self-concept clarity. Exploratively, we tested for effects of age and sex.

## 2 Methods

### 2.1 Participants

This project is part of the larger Leiden Self-Concept study. Global analyses on reflected vs. direct self-concept data (but not SMU) were reported previously (van der Cruijsen et al., 2019). From the initial 160 participants, several (N = 10) were excluded for the following reasons: excessive motion during the MRI scans (> 3 mm in one or more volumes in any translation or rotation direction, N = 8), not completing the MRI session (N = 1) and a technical error (N = 1). The final sample contained 150 participants (70 boys, 80 girls) between 11–21 years (M = 15.7, SD = 2.9). Longitudinal analyses were performed on questionnaire data from timepoint 2 (T2; M = 1.16 years after T1, SD = 0.074, N = 142) and timepoint 3 (T3; M = 1.10 years after T2, SD = 0.080, N = 137) (N reflects participants who also had complete T1 data). Missing data were handled in a pairwise manner: we always used all available data per participant. Exclusion criteria before participation were MRI-contraindications, left-handedness and a current or previous diagnosis of a neurological or psychiatric disorder. Scans were inspected by a radiologist and no clinically relevant findings were found. IQ was estimated with WISC-III (<16 years) or WAIS-III (>15 years) subtests Similarities and Block Design, and estimated IQ ranged between 80.0–137.5 (M = 110.30, SD = 11.06). Participants and parents of minors provided written informed consent. This study was approved by the Medical Ethics Committee of Leiden University Medical Center. FMRI-data published in this study are available on Neurovault and other data are available upon request. As part of the larger longitudinal Self-Concept study, all measures and hypotheses were pre-registered in the Open Science Framework (https://osf.io/8gc6x), but too late for this particular paper. The pre-registration details the mPFC-ROI definition used in the current study.

### 2.2 Questionnaires

#### 2.2.1 Social media use

**Fig. 1:**
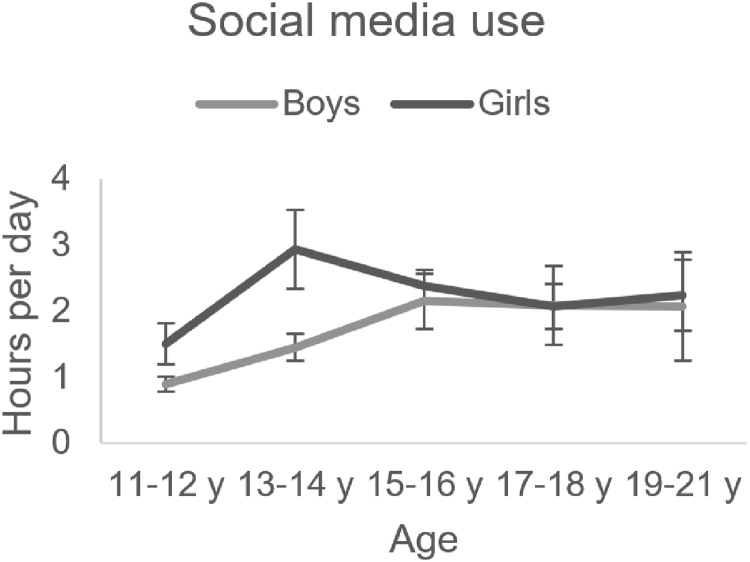
Frequency of social media use (#hours per day) per age-bin and per sex at T1. There was no effect of age or sex on social media use.

Similar to other studies (Orben and Przybylski, 2019b; Sampasa‐Kanyinga et al., 2018), SMU was measured by self-report. We asked participants: “How many hours a day do you typically spend on social media (e.g. Facebook, Twitter, Instagram)?” Participants indicated the number of hours (answer range from 0 to 24 discrete hours). Scores ranged between 0−12 hours per day (*M* = 2.06, *SD* = 1.87). SMU did not differ for boys and girls (*t*(134) = 1.44, *p* = .153) and was similar across ages (*r* = .10, *p* = .234) (Fig. 1).

#### 2.2.2 Positivity of self-concept

Positivity of the self-concept was measured with the fMRI-task, in which participants provided 60 self-judgements on academic, physical and prosocial traits. See section 2.3 for more details.

#### 2.2.3 Strengths and difficulties questionnaire

To assess clinical symptoms the self-report Dutch version of the Strengths and Difficulties Questionnaire (SDQ) was used (Goodman et al., 1998). This 25-item questionnaire measures the subscales emotional symptoms, conduct problems, hyperactivity/inattention, peer relationship problems and prosocial behavior. The SDQ has good internal reliability and validity (Van Widenfelt et al., 2003). Reliability scores for the current sample at each timepoint for each questionnaire and subscale are described in the Supplementary Materials.

#### 2.2.4 Prosocial behavior (self-report)

To measure self-reported prosocial behavior we used the prosocial behavior-subscale from the same Strengths and Difficulties questionnaire mentioned above.

#### 2.2.5 Self-concept clarity

The self-concept clarity scale (SCC) (Dutch version (Crocetti et al., 2008)) was used to assess adolescent’s self-concept clarity. This 12-item questionnaire consists items such as “I have a clear sense of who I am and what I am”. This scale has good internal reliability and predictive validity (Campbell, 1990), see Supplementary Materials for reliability at each timepoint in this sample.

### 2.3 FMRI self-concept task

**Fig. 2:**
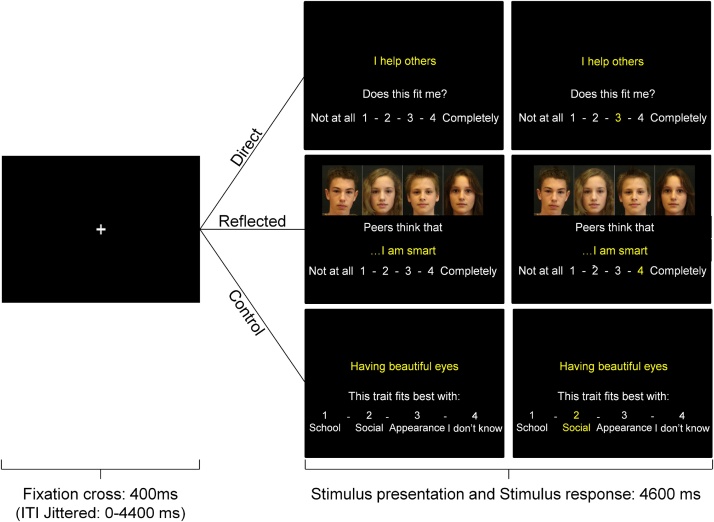
Trial-sequence for the self-judgement, reflected-peer-judgement and control tasks. In the self-judgement task, participants rated on a scale of 1-4 to what extent the traits described themselves. In the reflected-peer task, participants rated how they thought same-aged peers would rate them. During the control task, participants categorized the traits into one of the four options.

In the MRI scanner, participants performed a task in which they provided self-judgements on academic, physical and prosocial traits (Fig. 2). In the self-condition, participants rated themselves from their own perspective (e.g., “I am smart”). In the reflected-peer condition, participants rated themselves from their peers’ perspective (e.g. “Peers think that I am smart”). There were 60 matched trials in both conditions (20 academic, 20 physical, 20 prosocial, of which half positive and half negative traits, see Supplementary materials for examples). Participants rated whether traits applied to them on a scale from 1 (‘not at all’) to 4 (‘completely’). In the reflected-peer condition, pictures of unknown same-aged peers (morphed for anonymity) were presented to intensify the subjective experience of a social context and to remind participants to take their peers’ perspective while evaluating their traits. Additionally, there was a control-condition (20 trials) in which participants categorized traits into the best fitting category: (1) school, (2) social, (3) appearance, or (4) I don’t know. The three conditions were presented in separate blocks, with counterbalanced order between participants. Individual trials started with fixation (400 ms), followed by stimulus presentation (4600 ms). If participants did not respond during stimulus presentation, a ‘Too Late!’ screen appeared (1000 ms) (1.1 % of self-condition trials, 1.7 % of reflected-peer trials, 0.7 % of control trials. Trial-order and interstimulus jitter intervals (0–4.4 seconds) were optimized with Optseq.

For behavioral analyses, we calculated a score reflecting positivity of the self-concept. Responses on negative items were reverse coded and merged with positive items, for an overall global self-concept score (averaged across domains) and the three domains individually. To analyze the difference between self-judgements and reflected-peer-judgements, we calculated the difference score between responses to the same trait from the self-trial and reflected-peer trial (e.g. “I am smart” minus “My peers think I am smart”) and subsequently calculated the average difference across all items.

### 2.4 Data analyses

MRI-scan parameters are described in the supplementary materials. The MRI-scans were analyzed with SPM8 (Wellcome Department of Cognitive Neurology, London). Preprocessing steps were correction for slice-timing acquisition and rigid body movement, spatial normalizaton to MNI305 templates, spatial smoothing (6 mm). For first-level analysis, fMRI-timeseries were modelled as events with zero-duration. Modeled events were “Self-Academic-Positive”, “Self-Academic-Negative”, “Self-Physical-Positive”, “Self-Physical-Negative”, “Self-Prosocial-Positive” and “Self-Prosocial-Negative”, and the same events for the reflected-peer-condition, as well as “Control” for the control condition. Six motion-regressors were added (in addition to excluding participants who moved >3 mm). Motion during the task was correlated with age (*r*=-.27, *p* = .001). For group-level analyses, we computed whole-brain regressions for the contrasts Self>Control, Reflected-peer>Control and Reflected-peer>Self, with SMU and age as regressors (FDR cluster-level correction (*p* < .05) at an initial uncorrected threshold of *p* < .001). To investigate our mPFC-hypotheses, we used Marsbar to create a region-of-interest (ROI): an 8mm sphere based on a meta-analysis on mPFC during self-processing (Denny et al., 2012) (centre-of-mass: x=-6, y = 50, z = 4). Further analyses were carried out in R 3.6.1 and SPSS 22. We used linear regression analyses (controlling for age by adding age as first step in regression analyses, and after testing for the assumptions of linear regression). We also investigated whether sex moderated the relation between SMU and self-concept, using the PROCESS macro in SPSS (Hayes, 2012). Details of the mixed-model longitudinal analyses are described in the supplementary materials.

## 3 Results

### 3.1 Social media and self-concept positivity

**Fig. 3:**
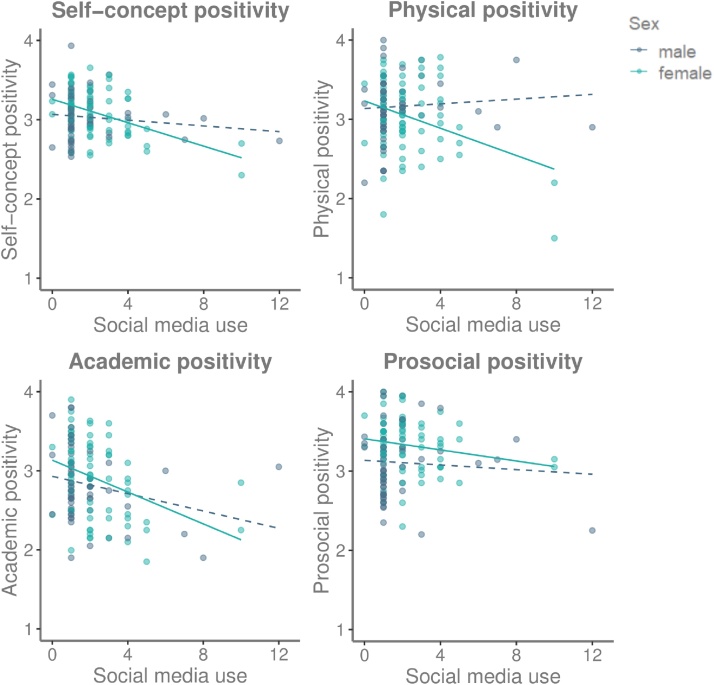
Scatterplots illustrating the relationships between social media use (#hours per day) and positivity of the self-concept, with separate regression lines for boys and girls. The different graphs show the relations for overall self-judgement positivity (across the 3 domains), and for physical, academic and prosocial self-judgement positivity individually.

We first tested whether SMU was linked to overall self-concept positivity as measured with the behavioral results of the fMRI-task. Hierarchical regression-analyses (age entered as first step as control variable, SMU as second step) revealed that SMU was correlated with less positive overall self-concept (*F*(2,133) = 6.06, *p* = .003, *R*^2^ = .08; *β*=-.29, *p* = .001). Sex was a moderator in this relationship (*F*(4,131) = 5.11, *p* < .001); there was a negative effect of SMU in girls (*F*(2,73) = 8.81, *p* < .001), *R*^2^ = .20; *β*=-.44, *p* < .001) but not boys (Fig. 3).

Next, we explored whether the relation between SMU and self-concept was different for the academic, physical and prosocial domain. We performed a repeated-measures ANCOVA with self-concept positivity in the three domains as within-subjects variables, SMU as continuous between-subjects variable and age as covariate. This analysis yielded a significant domain*SMU interaction (*F*(10,124) = 3.51, *p* < .001). Follow-up regression-analyses per domain revealed that adolescents with more SMU showed less positive academic self-concept (*F*(2,133) = 6.98, *R*^2^ = .10; *β*=-.31, *p* < .001, Fig. 3). For the physical domain, a moderation analysis revealed that sex moderated the relation between SMU and physical self-concept (*F*(4,131) = 3.66, *p* = .007), such that SMU was related to less positive physical self-concept in girls (*F*(2,73) = 4.73, *p* = .012, *R*^2^ = .12; *β*=-.33, *p* = .004) but not boys (Fig. 3). There was no relation between SMU and prosocial self-concept.

### 3.2 Self vs. reflected-peer-judgements

**Fig. 4:**
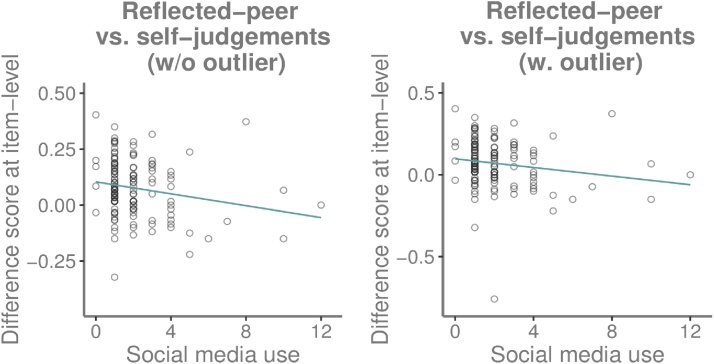
Scatterplots showing the relation between social media use (#hours per day) and the difference score between self-judgements and reflected-peer-judgements at item-level (e.g. “I am smart” minus “My peers think I am smart”). Left figure: without outlier, right figure: with outlier (>3x interquartile range). A difference score of 0 indicates no difference from the reflected-peer and self-perspective. Scores>0 indicate higher ratings from the self-perspective, scores<0 indicate higher ratings from the reflected-peer-perspective.

Next, we considered potential mechanisms in the relation between SMU and self-concept positivity. We tested whether frequent SMU was linked to reduced disagreement between self-judgments and reflected-peer-judgements in the fMRI-task (measured by calculating the difference score between self-judgements/reflected-peer-judgements for the same item (see Methods)). A regression-analysis (age-controlled) indicated that increased SMU related to less difference (visualized in Fig. 4) between self/reflected-peer-judgements (*F*(2,132) = 3.37, *p* = .038, *R*^2^ = .05; *β* = -.21, *p* = .016).

For this analysis, one outlier for the self-reflected difference (>3x interquartile range) which influenced the effect was removed, see Fig. 4 for an illustration with and without outlier. Sex did not moderate this relationship.

### 3.3 Social media use and neural mechanisms of self-concept

To investigate the connection between SMU and mPFC-activity, we used an a priori mPFC-ROI. We also performed whole brain-analyses with SMU and age as regressors for Self > Control, Reflected-peer > Control and Reflected-peer > Self, but there were no whole brain-clusters that survived FDRc-correction.

**Fig. 5:**
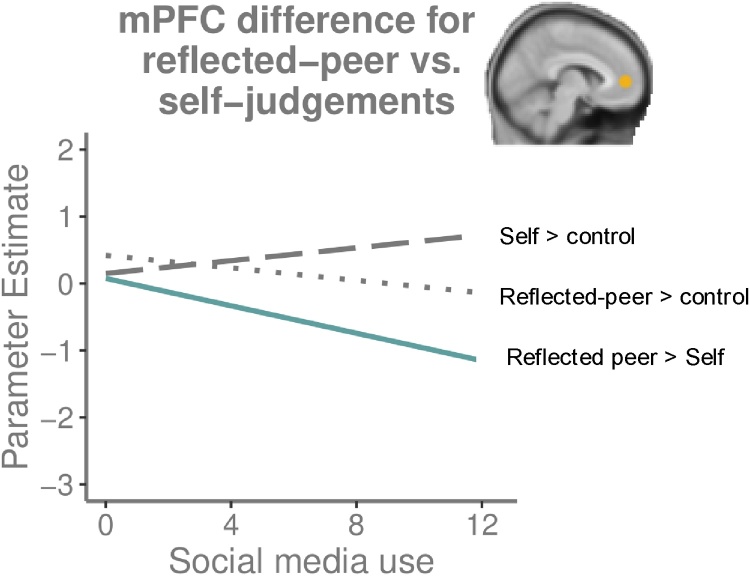
MPFC-ROI-activity for the predicted values for contrast Reflected-peer > Self. The x-axis shows social media use (#hours per day). The y-axis shows the predicted parameter estimates. A parameter value of 0 indicates no difference in mPFC-activity between reflected-peer-judgements and self-judgements, scores>0 point to relatively more activity during reflected-peer-judgements and scores<0 indicate relatively more activity during self-judgements. The predicted parameter estimates for the contrasts self-judgements > control and reflected-peer-judgements > control are also added to the figure.

To test our hypothesis that SMU is associated with increased self/reflected-peer-similarity in mPFC-activity, we examined mPFC ROI-activity for the contrast Reflected-peer > Self. A difference score of 0 would indicate completely similar activity. A regression-analysis (age as first step, SMU as second step) revealed that SMU was related to less mPFC-activity, *F*(2,133) = 4.284, *p* = .016, *R*^2^ = .061; *β* = -.205, *p* = .017, Fig. 5). Inspecting Fig. 5 reveals that heavier SMU corresponded to less mPFC-activity for reflected-peer-judgements and more for self-judgements. We also included the predicted values for Self > Control and Reflected-peer > Control to the figure for reference.

**Fig. 6:**
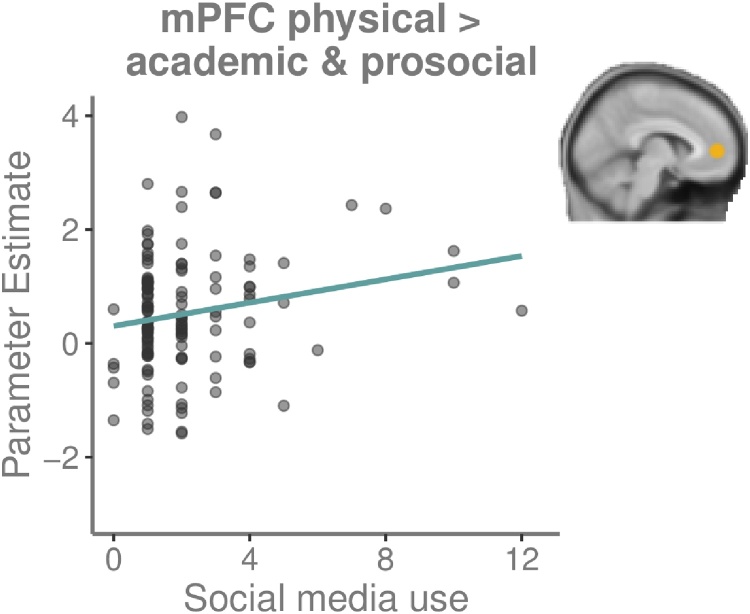
Scatterplot showing the relation between MPFC-ROI-activity for the contrast Physical > Academic & Prosocial self-judgements and social media use (#hours per day).

To examine whether the relation between SMU and mPFC-activity showed domain-specific effects, we performed further exploratory analyses for the contrast Reflected-peer > Self per domain. We tested whether relative activity during physical self-judgements compared to academic and prosocial self-judgements was related to SMU, and did the same for academic vs. physical and prosocial judgements (given the behavioral relations between SMU with physical and academic judgements). We calculated the contrasts Physical > Academic & Prosocial, and Academic > Physical & Prosocial in the mPFC-ROI. A regression-analysis (age as step 1, SMU as step 2) revealed that increased SMU was related to enhanced mPFC-activity for physical compared to academic and prosocial judgements (*F*(2,133) = 4.63, *p* = .011, *R*^2^ = .065; *β* = .17, *p* = .045) (Fig. 6). Sex did not moderate this relation. A regression for Academic > Physical & Prosocial indicated that increased SMU related to lower activity for academic compared to physical and prosocial judgements (*F*(2,133) = 4.43, *p* = .014, *R*^2^ = .062; *β* = -.19, *p* = .024), which fits with the prior physical analysis.

### 3.4 Longitudinal associations

We additionally tested for longitudinal relations between SMU, self/reflected-peer-difference and mPFC-activity with potential positive and negative outcomes. At T2 and T3 (each ∼1.1 years later), questionnaire data was available for SMU, clinical symptoms (SDQ subscales emotional, conduct, hyperactive and peer problems), prosocial behavior and self-concept clarity. First, we used longitudinal mixed-effects models (nlme package in R) to analyze whether SMU was related to these measures over time (at T1, T2 and T3). SDQ/self-concept clarity were dependent variables in separate analyses, SMU was predictor, and age and sex were control variables. There was no age effect in social media use across the three timepoints (*β* = -.02, *p* = .705), in line with the cross-sectional effects at T1 described earlier. Given the large number of dependent variables (6), we used Bonferroni correction with α = .05/6 = .0083.

Results of the multilevel analyses across three timepoints indicated that SMU showed no longitudinal relation with emotional, hyperactive or peer problems, prosocial behavior or self-concept clarity (correcting for age and sex). There was a longitudinal relation between SMU and enhanced conduct problems (*β* = .19, *p* < .001, corrected for age and sex) (Supplementary Table S1, Supplementary Fig. S1). Adding an interaction term for age*SMU and for sex*SMU yielded no significant interactions (Supplementary Table S2). We performed follow-up simple regression analyses to probe the direction of the effect (i.e., does social media use predict future conduct problems or do conduct problems predict future social media use?). However, follow-up simple regression analyses indicated that T1 SMU did not predict T2 conduct problems (controlling for age, sex, T1 conduct problems), nor did T1 conduct problems predict T2 SMU. This suggests that this relationship is not robust and shows no clear direction.

Self/reflected-peer difference scores and mPFC-activity were both based on the self-concept fMRI-task and only available at T1, therefore we only used regression-analyses (controlled for age, sex and baseline SDQ/self-concept clarity at T1) to test whether variables at T1 predicted outcomes at T2/T3. Self/reflected-peer-difference was negatively related to conduct problems at T2 (controlled for T1 conduct problems) (*F*(4,137) = 18.16, 4.31, *p* < .001, *R*^2^ = .35; *β* = -.224, *p* = .002, Fig. S2), but was no longer significant when predicting T3 conduct problems. Self/reflected-peer-difference was not related to other outcomes. Finally, mPFC-activity for Reflected-peer>Self-judgements was not related to any outcome variables.

## 4 Discussion

We investigated behavioral and neural mechanisms underlying the relation between self-reported social media use and self-concept. Our results revealed that SMU was associated with 1) less positive self-concept, driven by less positive physical self-concept (girls) and academic self-concept (boys and girls), 2) lower difference scores between self-judgements and reflected-peer-judgements, 3) heightened mPFC-activity during self-judgements compared to reflected-peer judgements, and 4) elevated mPFC-activity for physical compared to academic and prosocial self-judgements. Longitudinal analyses indicated no convincing relation between SMU and self/reflected-peer-difference with future clinical symptoms, prosocial behavior or self-concept clarity.

### 4.1 Self-concept from own vs. reflected-peer perspective

Our results suggest that adolescents with heavier SMU showed less difference in the positivity of self-judgements from their own and their reflected-peer-perspective, i.e. ratings such as ‘I look good’, vs. ‘My peers think I look good’ were less different. During the reflected-peer task, images of unknown same-aged peers were presented, to enhance the subjective impression of a peer audience. The reflected-peer task might be similar to experiences in a social media environment, as prior research revealed that when on social media, people are highly conscious of their followers and try to imagine the perspective of their audiences on their posts (Marwick and Boyd, 2011). Social media also enable more salient and frequent social feedback compared to offline life (Valkenburg, 2017) which may make it more evident to adolescents how peers feel about them. These processes may therefore enable this reduced differentiation between adolescents’ own judgements and how they think peers will judge them. Speculatively, adolescents with less SMU may have a more ‘autonomous’ self-concept. They may be less concerned with how peers feel about them and have a more independent view of who they are. Nevertheless, perceived opinions from other people than peers such as parents, siblings or teachers might be important as well. This may be an especially interesting hypothesis for future developmental research as incorporating the opinions of others into the self-concept has always been considered an important aspect of self-concept development (Harter, 2015). Perhaps the rise of social media use will have an influence on how the self is constructed, but further longitudinal research is needed to fully uncover this question.

In general, we found that SMU was related to less positive self-concept, especially in girls, which is in line with some prior studies (Blomfield Neira and Barber, 2014; Woods and Scott, 2016). When analyzing domain-specific relations, heavier SMU related to less positive academic self-concept and physical self-concept (the latter especially in girls). A potential contributor to this effect is that there is a bias in sharing positive information on social networks (Reinecke and Trepte, 2014). This may lead adolescents to compare themselves with idealized versions of their peers and they may therefore expect to be evaluated less positively by peers. Especially when reflected peer-opinions are more integrated into the self-concept, this could lead to a less positive self-concept. Other research already demonstrated that upwards social comparisons lead to lower global self-esteem (Vogel et al., 2014) and physical self-esteem (De Vries and Kühne, 2015), with some indications that these effects may be stronger for female participants (Vogel et al., 2014). A parallel process of upwards comparison might play a role in academic self-concept, but increased time on social media might also distract from schoolwork, which may in turn affect academic performance and self-concept. However, this will need to be confirmed in future studies.

Importantly, the reverse relations might also be true: adolescents with less positive self-concept might spend more time on social media, and prior findings already indicated that social self-esteem predicted later SMU (Valkenburg et al., 2017). Another possibility is that there are confounding variables in which the high-SMU users differ from moderate users, which drives both their high SMU and negative self-concept (Twenge, 2019a). It should be noted that our study was mainly suitable for investigating behavioral and neural mechanisms, and large-scale longitudinal population studies are better suited to answer the general question whether SMU is associated with self-concept or developmental outcomes. Longitudinal studies also provide opportunities for mediation analyses which are less affected by under- and overestimation of mediation-parameters (O’Laughlin et al., 2018). Prior large-scale studies on associations between SMU and wellbeing more broadly already exist but the effects and practical significance are highly debated (Orben and Przybylski, 2019b; Twenge, 2019b).

### 4.2 SMU and medial prefrontal cortex activity

To date, prior research has not investigated whether SMU is related to the neural mechanisms of self- and reflected-peer processing. Given the reduced difference between self/reflected-peer-ratings uncovered in the behavioral analyses, we expected a mirroring pattern in mPFC-activity. Instead, we discovered that mPFC is relatively more active during self-ratings compared to reflected-peer-ratings with heavier SMU. Potentially, SMU is related to enhanced specialization in mPFC for self vs. reflected ratings. Inspecting mPFC-activity for self-ratings and reflected-ratings separately indeed highlights more similar activity for self/reflected-peer-ratings with low SMU, and larger differentiation with high SMU. SMU might lead to intensified practice in reflecting about the self and imagining how others feel about you, which may contribute to specialization in prefrontal cortex (Johnson, 2011). Alternatively, given the behavioral results indicating reduced difference between self/reflected-peer-ratings, it might be that with high SMU, the process of imagining how peers reflect about you requires less effort and is more automatic. This might result in relatively less mPFC-activity for reflected-peer > self-judgements, as less effort has been hypothesized to be expressed through lower PFC-activity (Luna et al., 2010). These are all preliminary hypotheses that should be confirmed in future studies.

We additionally explored whether SMU showed a connection with domain-specific mPFC-activity during self-judgments. These analyses uncovered that for participants with heavier SMU, mPFC was more active during physical self-judgements compared to academic and prosocial self-judgments. This suggests that mPFC, the central region involved in self-processing, is especially sensitive to physical self-judgements with more SMU. Tentatively, this might be related to the emphasis social media place on physical appearance (de Vaate et al., 2018), but this needs to be confirmed in future research.

### 4.3 Longitudinal relations

An important question is whether social media use and incorporating peer’s views into the own self-concept is adaptive or maladaptive in self-concept development. Incorporating others’ views into the self-concept has been posited as a normal and important milestone in self-concept development (Harter, 2015). Potentially, it could encourage accelerated development of self-concept clarity, better perspective-taking and more prosocial behavior. However, it could also indicate excessive concern with others’ opinions about the self, which has been tied to clinical symptoms such as depression (Beck et al., 2001). To probe into this question, we used longitudinal follow-up questionnaire data collected approximately one and two years later. Mixed-model analyses indicated a longitudinal relation between SMU and higher scores on conduct problems, but no clear direction to this effect could be found. We also found no support for a longitudinal relation between SMU and emotional, hyperactive, and peer problems, self-concept clarity or prosocial behavior. Increased self/reflected-peer-integration (only available at T1) related to more conduct problems at T2, but was no longer related to conduct problems at T3. MPFC-activity for self > reflected-peer processing also showed no relation with these longitudinal outcomes. Overall, the current study indicated no convincing positive or negative longitudinal effects, similar to Orben and Przybylski (2019a). Important to note is that reliability for several subscales of the SDQ (conduct problems, peer problems and prosocial behavior) was low, therefore these findings must be interpreted with caution. Large-scale longitudinal studies and randomized intervention studies (Hunt et al., 2018) are needed to further unravel these effects. Furthermore, future work should also obtain objective measures of SMU, as self-reported internet use is an imperfect reflection of actual use (Scharkow, 2016). Finally, studies are starting to unravel that not only the time spent on social media, but also *what* they are doing is important to measure (Aalbers et al., 2019; Prinstein et al., 2020).

### 4.4 Conclusions

In conclusion, this study suggests that more frequent SMU is associated with a smaller difference between how adolescents rate themselves and how they think peers will rate them. Lower SMU was linked to more positive ratings from participant’s own compared to the reflected-peer-perspective. Importantly, this mechanism was also related to a less positive self-concept in girls. Moreover, mPFC showed higher activity for self- vs. reflected-peer processing and seemed especially sensitive to physical self-judgments in participants with frequent SMU. Although future research is still highly needed, interventions for negative self-concept might benefit from discussing social media use, the subjective experience of the imaginary audience and feeling constantly judged by peers, and the idealized online lives of peers which form an unfair comparison group to the developing self (de Vaate et al., 2020).

## Data statement

The data that support the findings of this study are available on request from the corresponding author, s.peters@fsw.leidenuniv.nl.

## Declaration of Competing Interest

The authors declare no conflict of interest.
