# Supplementary material for: Social media use and the not-so-imaginary audience: Behavioral and neural mechanisms underlying the influence on self-concept
Source: Dev Cogn Neurosci. 2021 Jan 26;48:100921. doi: 10.1016/j.dcn.2021.100921 (PMC7848768; doi:10.1016/j.dcn.2021.100921)
Supplement: Supplementary file 1 [file mmc1.docx]

The data that support the findings of this study are available on request from the corresponding author, s.peters@fsw.leidenuniv.nl
